# Allied health professionals’ research capacity: open to interpretation?

**DOI:** 10.1186/s12913-023-09678-z

**Published:** 2023-06-14

**Authors:** Terry Cordrey, Elizabeth King, Owen Gustafson

**Affiliations:** 1 Oxford Allied Health Professions Research and Innovation Unit, Head of Therapies Office, Oxford University Hospitals NHS Foundation Trust, Oxford, OX3 9DU UK; 2 Centre for Movement Occupational and Rehabilitation Sciences, Faculty of Health and Life Sciences, Oxford Brookes University, Oxford, UK

**Keywords:** Research capacity, Research culture, Allied health professionals, Survey

## Abstract

Allied health professional research capacity and culture has been the focus of growing research interest of late. The recent study by Comer et al. represents the largest survey of allied health research capacity and culture to date. We congratulate the authors on this work and would like to raise some discussion points in relation to their study.

The authors have interpreted their research capacity and culture survey results using cut-off values to indicate a degree of adequacy in relation to perceived research success and/or skill level. To our knowledge, the constructs of the research capacity and culture tool have not been validated to an extent that would enable such an inference to be made.

Comer et al. describe perceived individual research success and/or skill as adequate, but the rating of skills in areas necessary for the conduct of original research, such as writing research protocols, ethics submissions, securing funding, and writing for publication range from median scores one to three, which is considered ‘less than adequate’ on the interpretation scale used by the authors.

The survey results for the individual and organisational domains reported in Comer et al. are comparable to other similar studies. However, they uniquely conclude research success and/or skill to be adequate in both domains, which is contrary to the interpretation of the other studies.

The interpretation of allied health professional research success and skill offered by Cromer et al. differs from studies with similar results and is contrary to previous reports of insufficient research capacity in terms of research trained and active practitioners within these professions in the UK.

## Main text

We would like to congratulate Comer et al. for undertaking the largest survey to date evaluating the research capacity and culture of Allied Health Professionals (AHP) in the United Kingdom national health service [1]. We would like to raise a few points in relation to this study.

First, Comer et al. use the Research Capacity and Culture (RCC) tool, which is a valid and reliable instrument to measure perceptions of research success and/or skill across individual, team and organisational domains in healthcare [2]. Scores from the questions in each of the three domains were aggregated and categorised into “less than adequate”, “adequate” or “more than adequate” based on a similar method used in a previous study [3]. The study cited by Comer et al. to guide their method uses the terms “low” (mean < 4), “medium” (4–6.99), and “high” (7 or above) to describe research success and/or skill, but there is no reported description of adequacy in relation to these cut off values. Assigning descriptive terms to values of the RCC scale, such ‘low’, ‘medium’, and ‘high’ has been reported in several studies, but use of the term ‘adequate’ as an adjective to interpret skill or success level from the scale is unprecedented [3–5]. In describing the scale values in this way, the authors of this study do not outline or cite the construct validation process that enables the RCC tool to be categorised as such. It has been previously identified that the RCC tool does not provide cut off values to enable a meaningful interpretation of the score beyond the original definitions of the scale (1 = no success/skill; 10 = highest possible success/skill) [6].

**Fig. 1:**
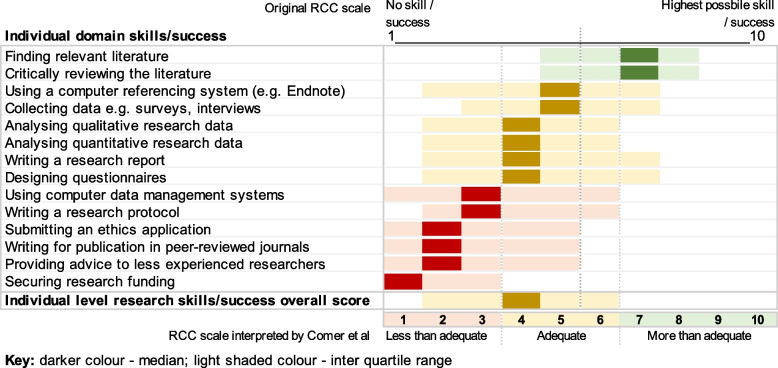
Individual domain results from Comer et al. in the context of RCC scale interpretation

Second, using the categorisation method described above, the authors conclude that AHPs perceive their individual research success and/or skill to be adequate, and emphasise that the barriers to integration of AHP research into clinical practice are at the team level. However, upon evaluation of the individual AHP data they report (Fig. 1), skills considered critical to the conduct of research, such as securing funding, writing a research protocol, submitting to an ethics committee, and writing for peer-reviewed publication are rated lowly ranging from median scores of one to three. This would be considered less than adequate using the same categorisation method deployed by the authors. This observation is not explored in the published manuscript by Comer et al., but it is identified in a preliminary report of the same data set by the authors, who, at that point, concluded AHPs to be confident in literature searching and critiquing, but not in other research skills [7].

The authors’ interpretation of individual AHP research success and/or skill as adequate is contrary to the conclusion of other recent studies using the RCC tool to explore AHP research capacity [4, 5, 8–10]. Despite reporting similar scores to Comer et al., these studies conclude a lack of sufficient individual AHP success and/or skill across areas necessary to conduct research. This is illustrated in Table 1 where AHPs appear to be relatively successful consumers of research, but less so producers of their own research. This distribution of skill level may well reflect the greater need for practising AHPs to translate evidence rather than produce it. However, this has yet to be established and inferring an adequate skill level across the broad range of skills in this domain may obscure the need for targeted strategies and investment to develop capability in these areas.

**Table 1:** AHP individual success and/or skill median scores (interquartile range) from recent research capacity and culture studies

Individual domain skills	Comer et al., 2022 [1]	Alison et al., 2017 [4]	Cordrey et al., 2022 [8]	Crombie et al., 2021 [9]	Frakking et al., 2021 [6]	Matus et al., 2020 [5]	Matus et al., 2019 [5]
Study details	National cross-sectional survey, UK	Prospective survey, Australia	Mixed methods single centre, England	Cross-sectional survey with comparison, Australia	Cross-sectional observational, Australia	Cross-sectional survey, Australia	Cross-sectional observational, Australia
Finding relevant literature	7 (5–8)	7 (6–8)	6 (5–8)	7 (6–8)	7 (5–8)	7 (5–8)	7 (6–8)
Critically reviewing the literature	7 (5–8)	7 (6–8)	6 (5–7.75)	7 (6–9)	6 (3–8)	6 (5–7)	7 (5–8)
Using a computer referencing system (e.g. Endnote)	5 (2–7)	6 (3–8)	4 (1–6)	5 (3–7)	5 (2–7)	5 (2–7)	6 (3–7)
Writing a research protocol	3 (2–6)	5 (3–7)	2 (1–5)	4 (3–6)	3 (1–6)	3 (2–5)	4 (2–7)
Securing research funding	1 (1–3)	2 (1–4)	1 (1–2)	2 (1–4)	1 (1–3)	2 (1–2)	3 (1–4)
Submitting an ethics application	2 (1–5)	3 (1–6)	1 (1–3)	3 (1–4)	2 (1–3)	2 (1–5)	3 (1–6)
Designing questionnaires	4 (2–7)	5 (3–7)	5 (2.5–7)	6 (4–8)	4 (1–7)	5 (2–6)	5 (3–6)
Collecting data e.g. surveys, interviews	5 (3–7)	6 (5–8)	5.5 (3–7)	6 (5–7)	5 (3–7)	5 (3–7)	6 (4–8)
Using computer data management systems	3 (1–6)	5 (2–7)	3 (1–5.75)	3 (2–5)	4 (2–6)	3 (2–6)	5 (2–7)
Analysing qualitative research data	4 (2–6)	5 (2–7)	4 (1–5)	4 (3–6)	3 (1–6)	3 (2–5)	4 (2–7)
Analysing quantitative research data	4 (2–6)	5 (2–7)	4 (1–5)	4 (3–6)	4 (1–7)	3.5 (2–6)	4 (2–7)
Writing a research report	4 (2–7)	5 (3–7)	3 (1.5–6)	5 (3–7)	3 (1–6)	3 (2–6)	5 (2–7)
Writing for publication in peer-reviewed journals	2 (1–5)	4 (2–7)	2 (1–5)	3 (2–5)	2 (1–3)	2 (1–5)	3 (2–6)
Providing advice to less experienced researchers	2 (1–5)	3 (1–6)	2 (1–4)	3 (2–5)	2 (1–3)	2 (1–4)	3 (2–6)
Study interpretation of Individual skill/success	Adequate individual research success /skills	Limited skills in individuals to carry out research	Lack of individual skills across the whole research process	No change in individual scores between surveys (4 years). High scores in early research skills	Limited individual research capacity	Individual skills are highest in the preliminary stages of the research process	Moderate to high skills in ‘early’ research processes

Third, the authors conclude that AHPs perceive organisational research success and/or skill to be adequate. However, similarly to the individual domain, the success and/or skill level in this domain is rated low in areas essential to the growth and development of AHP research, such as support for research training, resources to carry out research, and availability of career pathways in research. The latter point is particularly important to consider since the lack of clinical academic and research career pathways for AHPs in the UK has been widely acknowledged [11]. The absence of an agreed standard or threshold by which to judge organisational research capacity and skill makes it difficult to determine a level of adequacy [12]. Further work to understand what constitutes an adequate level of organisational research capacity and skill beyond that perceived by its employees would potentially help direct future research capacity building for AHPs.

Lastly, research capacity building (RCB), defined as “a process of developing sustainable abilities and skills enabling individuals and organisations to perform high quality research” [13], is central to the scope and vision of the Allied Health Professions Research and Innovation Strategy for England [14]. In conducting the largest survey of AHP research capacity and culture to date, Comer et al. report data comparable to several previous studies (Table 1), which concludes that AHPs lack the full range of skills, available support, and career infrastructure to undertake research effectively. Comer et al. are unique amongst these studies in concluding individual and organisational research success and/or skill level to be adequate. The absence of evidence and objective measures to determine an adequate skill and success level in AHP research capacity arguably enables this outlying interpretation [15]. Until progress is made in this respect, it is important that findings from all studies in this field are considered and serve as a proxy indicator of the current status to ensure that individual AHPs and the organisations in which they work are not overlooked in future RCB strategies.

## Conclusion

This correspondence article congratulates Comer et al. on their UK-wide cross-sectional survey of allied health professional research capacity and culture. It goes on to raise discussion points in relation to 1) the use of cut-off values to interpret the research capacity and culture tool, 2) the interpretation of individual research success and skill as adequate despite low perceived success and/or skill in areas necessary to conduct research, and 3) the interpretation of individual and organisation research success and/or skill as adequate, which is contrary to the conclusion of other studies with similar survey results.

## Data Availability

All data and material for this correspondence article is included within the publication.

## Abbreviations

AHP: Allied Health Professional
RCC: Research Capacity and Culture
UK: United Kingdom
RCB: Research Capacity Building
